# Neurological diagnostic tests for patients with and without delirium: a prospective observational study

**DOI:** 10.1007/s11357-024-01246-5

**Published:** 2024-06-25

**Authors:** Noémie Waefler, Imen Abid, Victor Montaut, Jacques Donzé, Hervé Zender, Gregor John

**Affiliations:** 1 Department of Internal Medicine, Neuchâtel Hospital Network, Rue de la Maladière 45, CH-2000 Neuchâtel, Switzerland; 2 Division of Internal Medicine, Inselspital, Bern University Hospital, Bern, Switzerland; 3 Department of Medicine, University Hospital of Lausanne, Rue de Bugnon 21, CH-1011 Lausanne, Switzerland; 4 Brigham and Women’s Hospital, Harvard Medical School, Boston, MA USA; 5 Department of Medicine, Neuchâtel Hospital Network, Rue du Chasseral 20, CH-2300 La Chaux-de-Fonds, Switzerland; 6 Department of Acute Medicine, Geneva University Hospitals (HUG), Gabrielle-Perret-Gentil 4, CH-1205 Geneva, Switzerland; 7 Department of Internal Medicine, Geneva University Hospitals (HUG), Gabrielle-Perret-Gentil 4, CH-1205 Geneva, Switzerland; 8 University of Geneva, Rue Michel Servet 1, CH-1211 Geneva, Switzerland

**Keywords:** Delirium, CT scan, MRI, Electroencephalography, Lumbar puncture

## Abstract

Since most of the precipitating factors of delirium are not due to neurological disorders, neurological diagnostic tests (NDTs) may be of limited value. We hypothesized that delirium has a high burden of NDTs with a low diagnostic yield. All patients admitted to the internal medicine wards of a single secondary teaching hospital between November 2019 and January 2020 were eligible. Within the first 48 h of their admission, they had a formal evaluation by a neuropsychologist to screen for presence of delirium. NDTs (brain MRI, brain CT, electroencephalography (EEG), and lumbar puncture) performed during the hospital stay were compared between patients with and without delirium using a logistic regression model stratified by a propensity score. The proportions of diagnostic yield (acute anomalies that changed the treatment management) provided by each type of examination were compared. Of 217 patients included, 19/32 patients (61%) with delirium had one or more NDTs, compared to 48/185 (26%) without delirium (adjusted OR 2.7; 95%CI 1.1–6.7; *p* = 0.027). The proportions of NDT results affecting management for patients with and without delirium were 13 and 20% for brain CT scans (*p* = 0.71), 29 and 38% for brain MRI (*p* = 0.99), and 20 and 10% for EEGs (*p* = 0.99), respectively. The higher proportion of NDTs performed on patients with delirium was associated with a low diagnostic yield, although not statistically different from those performed among inpatients without delirium. There is a need for restrictive, evidence-based guidelines to help with the work-up for patients with delirium.

**Supplementary Information:**

The online version contains supplementary material available at 10.1007/s11357-024-01246-5.

## Introduction

Delirium is frequent among adult inpatients, with a prevalence at admission ranging from 11 to 33% [1–3], and an incidence during the hospital stay of 3 to 56% [4–7]. It has been associated with a higher risk of falls, increased hospital length of stay (LOS), worsening cognitive and functional status, and mortality [8–10].

The most common triggers of delirium in hospitalized patients are acute medical illness (e.g., infection, electrolyte imbalance, renal failure), trauma (with fractures or head injury), surgery, dehydration, and psychological distress [11, 12]. Drug toxicity (e.g., from anticholinergic medications, sedative-hypnotics, analgesics, digoxin, valproate, or steroids) comprise 30% of all cases [13]. Since the usual triggers are not neurological infections or diseases, neurological diagnostic tests (NDTs) like cerebral imaging, electroencephalogram (EEG), or lumbar puncture (LP) are often unhelpful. Theisen-Toupal et al. found only 2.7% of positive diagnoses using computed tomography (CT) performed on patients admitted for delirium [14]. Similarly, Warshaw et al. showed that 99% of patients with febrile delirium who underwent a LP did not have a central nervous system infection [15]. Finally, although non-convulsive epilepsy is one of the most frequently missed diagnoses in patients presenting with an altered mental status [16], prospective studies of an EEG’s utility in delirium are lacking, and an EEG is unnecessary for diagnosing delirium. Thus, these NDTs are expensive and often have a low diagnostic yield.

Several guidelines have proposed criteria and general considerations for using NDTs in the workup for delirium’s etiology [16–20]. The NICE guidelines also suggest looking for underlying causes if delirium is not resolved using the recommended pharmacological and non-pharmacological methods [20]. However, red flags are frequent, nonspecific or hardly excluded in older adults admitted with delirium (e.g., recent history of falls, altered level of consciousness), and delirium can persist for days or weeks even if the trigger has been corrected. Thus, cerebral imaging, an EEG, or LP are performed based on clinician’s expertise. The proportion of NDTs performed on patients admitted to acute internal medicine wards has seldom been reported. We hypothesized that delirium has a high burden of NDT.

We aimed to explore whether NDTs were used differently among patients with and without delirium. Secondarily, we aimed to describe the clinical management of delirium and its associated risks of readmission or death.

## Methods

We conducted a prospective observational cohort study of the patients admitted to the internal medicine ward of a single secondary teaching hospital between November 1, 2019, and January 6, 2020. NDTs performed during the hospital stay and death or readmission within 90 days of the index hospitalization were recorded. Written informed consent was obtained from the patient or their closest relative if they were unable to consent themselves. The Human Research Ethics Committee of the Canton of Vaud approved the study (CER-VD, *2019-01428*). Procedures were performed according to Good Clinical Practice standards and the Declaration of Helsinki and were reported using the STROBE guidelines [21].

### Participants

Patients aged 18 or more who were admitted to an internal medicine ward department during the study period were included consecutively. Patients needing emergent care, not speaking French, with a planned LOS of fewer than 3 days, or who did not undergo formal evaluation of their delirium status by a neuropsychologist were excluded.

### Outcomes and measurements

The primary outcome was the proportion of patients who underwent an NDT. An NDT was defined as any EEG, brain CT scan, brain magnetic resonance imaging (MRI), or LP performed during the hospital stay.

The secondary outcomes were the four individual NDTs, the proportion of abnormal results found via NDTs, other examinations and elements of delirium management (number of blood punctures during the first 7 days of hospital admission, discontinuation of drugs potentially associated with delirium, use of chemical restraint, use of physical restraint), LOS, 90-day mortality, or hospital readmission.

Based on the final reports signed by the appropriate specialists (radiologists or neurologists), abnormal NDT results were classified as minimal/non acute changes when they had no impact on medical management (e.g., brain atrophy) or as acute changes when they helped in the diagnosis or impacted the patient’s management (e.g., acute stroke, subdural hematoma, focal irritability on EEG). This dichotomy was made by consensus between three authors (VM, AI, and GJ). Drugs potentially associated with delirium were opioids, benzodiazepines, and/or treatments with known anticholinergic side effects. Chemical restraint was defined as the introduction of a new neuroleptic or benzodiazepine prescription. Physical restraint was defined as any device used to limit the patient’s motion.

Delirium was assessed during a 15–30-min face-to-face interview with a neuropsychologist performed within the first 48 h of admission. The diagnosis of delirium was defined according to the Diagnostic and Statistical Manual of Mental Disorders (DSM)-V criteria and the CAM method [22]. In cases involving an undefined confusional state, doubts were resolved by consulting with two other neuropsychologists.

Patients were only seen once after their inclusion in order for information to be collected by the study team. Data were gathered using patients’ electronic medical records comprising their past medical history, tests performed and their results, medication history, physical restraint, and ongoing treatment. Comorbidity burdens were summarized using the Charlson comorbidity index (CCI) [23].

Data on 90-day death and hospital readmission post-discharge were collected via the hospital’s electronic medical records. Neuchatel Hospital Network is the only public hospital in the region and covers almost every hospital admission. Hospitalization in other Swiss region or in private clinics was not captured in this data.

### Statistics

The cohort sample size was chosen to develop a delirium screening tool, published previously [24]. With a sample size of 217 patients, a prevalence of delirium of 15%, the a posteriori study power was more than 80% to demonstrate an absolute 30% difference in the NDTs between patients with and without delirium, and less than 60% for a 20% absolute difference.

For the primary analysis, we performed a stratified logistic regression, with NDTs as the dependent variables and delirium as the independent variable. Patients were stratified into four strata of a propensity score. Stratification using propensity scores avoids losing the information of unmatched patients (which is frequent in propensity score matching) or of patients not in the common support (when using propensity weighted analysis) [25]. The propensity score was constructed using a logistic regression model, with delirium as the dependent variable, and it included all the confounders associated with the outcome (NDTs) and the exposure (delirium) or variables associated with the outcomes alone (eTable1) [26]: age, use of neuroleptics, an oncological disease, urinary catheter, urinary incontinence, past stroke or transient ischemic attack, cognitive impairment, and atrial fibrillation. The propensity score stratification resulted in well-balanced factors between groups (eFigure 1). We performed a sensitivity analysis excluding all the controls who were diagnosed with delirium later in their hospital stay.

Secondary binary outcomes were also analyzed using logistic regression and stratified logistic regression models. We used a log-transformed LOS linear regression stratified for the propensity score to compare groups, since untransformed LOS were not normally distributed.

We used nonparametric descriptive statistics for general characteristics (medians and IQR). Comparisons of characteristics between groups were performed using chi-squared tests or Fisher tests, or Mann-Whitney tests where appropriate. Significance levels were set at 5%, and all analyses were performed using STATA software, version 17.0 (StataCorp LP, College Station, TX, USA).

## Results

**Fig. 1:**
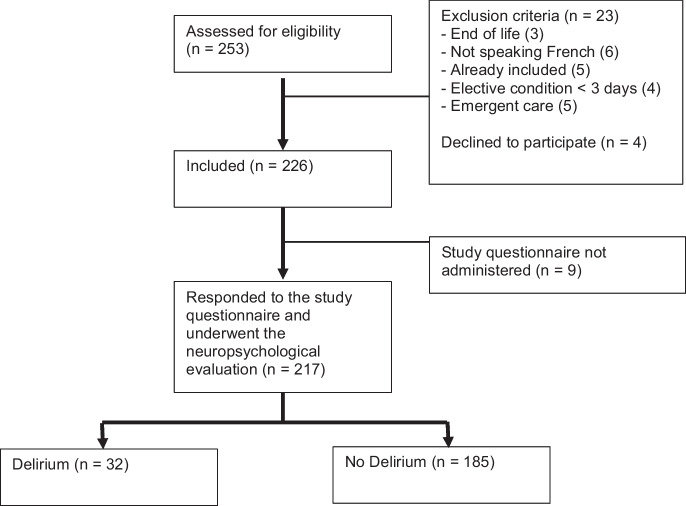
Study flowchart

Of the 253 patients screened, 217 were included (Fig. 1). The prevalence of delirium in the first 48 h of hospitalization was 14.3% (95%CI 9.6–19.0%). Patients with delirium were older had more frequent myocardial infarction, cognitive impairments, acute renal injuries, stroke, psychiatric comorbidities, rheumatic comorbidity, malnutrition, pre-admission neuroleptic use, pre-admission antidepressant use, urinary catheter placement, and a higher CCI (Table 1). Infection and drug side effects were the two most frequent precipitating factors of delirium. Only 5/32 patients (16%) experienced delirium due to a neurological trigger (eTable 2).

**Table 1:** Characteristics of patients with and without delirium

Characteristic	Cohort (*N* = 217)	With delirium (*N* = 32)	Without delirium (*N* = 185)	*P* value
Age (y), median (IQR)	75.9 (66.5–85.8)	84.0 (73.2–90.7)	75.2 (64.1–84.7)	<0.01
Male	109 (50.2%)	11 (35.5%)	98 (52.7%)	0.08
Admitted through ER	205 (94.5%)	31 (100%)	174 (93.5%)	0.22^a^
Non smoker	152 (70.0%)	24 (77.4%)	128 (68.8%)	0.71^a^
Current smoker	43 (19.8%)	5 (16.1%)	38 (20.4%)	
Former smoker	22 (10.1%)	2 (6.4%)	20 (10.7%)	
Charlson comorbidity index, median (IQR)	3 (2–5)	4 (3–6)	3 (2–5)	0.03
High blood pressure	140 (64.5%)	23 (74.2%)	117 (62.9%)	0.22
Myocardial infarct	53 (24.4%)	12 (38.7%)	41 (22.0%)	0.04
Atrial fibrillation	63 (29.0%)	11 (35.5%)	52 (28.0%)	0.39
Peripheral vascular disease	19 (8.8%)	2 (6.5%)	17 (9.1%)	0.99 ^a^
Heart failure	90 (41.5%)	16 (51.6%)	74 (39.8)	0.22
Stroke	43 (19.8%)	11 (35.5%)	32 (17.2%)	0.03 ^a^
Cognitive impairment				<0.01
Mild	44 (20.3%)	11 (35.5%)	33 (17.7%)	
Severe	39 (18.0%)	11 (35.5%)	28 (15.0%)	
Diabetes	49 (22.6%)	7 (22.6%)	42 (22.6%)	0.99 ^a^
Insulin	20 (9.2%)	3 (9.7%)	17 (9.1%)	
No insulin	29 (13.4%)	4 (12.9%)	25 (13.4%)	
COPD	40 (18.4%)	4 (12.9%)	36 (19.3%)	0.46 ^a^
Oncological disease				0.30 ^a^
Without metastasis	40 (18.4%)	6 (19.3%)	34 (18.3%)	
With distant metastasis	26 (12.0%)	1 (3.2%)	25 (13.4%)	
Mild liver disease	17 (7.8%)	2 (6.5%)	15 (8.1%)	0.91 ^a^
Severe liver disease	13 (6.0%)	1 (3.2%)	12 (6.4%)	
AKI				0.02 ^a^
1	53 (24.4%)	10 (32.3%)	43 (23.1%)	
2	10 (4.6%)	2 (6.4%)	8 (4.3%)	
3	5 (2.3%)	3 (9.7%)	2 (1.1%)	
CKD *0*	162 (74.7%)	18 (58.1%)	144 (77.4%)	0.12 ^a^
I	1 (0.5%)	0 (0%)	1 (0.5%)	
II	5 (2.3%)	1 (3.2%)	4 (2.2%)	
III	34 (15.7%)	9 (29.0%)	25 (13.4%)	
IV	15 (6.9%)	3 (9.7%)	12 (6.4%)	
V	-	-	-	
Rheumatological disease	78 (35.9%)	17 (54.8%)	61 (32.8%)	0.02
Anemia	80 (36.9%)	11 (35.5%)	69 (37.1%)	0.86
Psychiatric illness	71 (32.7%)	15 (48.4%)	56 (30.1%)	0.04
Alcohol consumption				
Active	38 (17.5%)	4 (12.9%)	34 (18.3%)	0.53 ^a^
Former	17 (7.8%)	1 (3.2%)	16 (8.6%)	
Drug abuse				0.99 ^a^
Active	9 (4.1%)	1 (3.2%)	8 (4.3%)	
Former	4 (1.8%)	0 (0%)	4 (2.1%)	
Malnutrition				0.01 ^a^
Mild	24 (11.1%)	5 (16.1%)	19 (10.2%)	
Moderate	31 (14.3%)	7 (22.6%)	24 (12.9%)	
Severe	25 (11.5%)	7 (22.6%)	18 (9.7%)	
Nasal tube feeding	8 (3.8%)	3 (10.3%)	5 (2.8%)	0.08
Urinary incontinence	48 (22.1%)	10 (32.3%)	38 (20.4%)	0.14
Nocturia	104 (47.9%)	7 (22.6%)	97 (52.1%)	<0.01 ^a^
Urinary catheter	25 (11.5%)	9 (29.0%)	16 (8.6%)	<0.01 ^a^
Surgery within the month	12 (5.5%)	5 (16.1%)	7 (3.8%)	0.02 ^a^
Pre-admission diuretics use	88 (40.5%)	13 (41.9%)	75 (40.3%)	0.87
Pre-admission opioids	59 (27.2%)	9 (29.0%)	50 (26.9%)	0.83 ^a^
Pre-admission neuroleptic use	36 (16.6%)	10 (32.3%)	26 (14.0%)	0.02 ^a^
Pre-admission antidepressant use	49 (22.6%)	14 (45.2%)	35 (18.8%)	<0.01
Pre-admission benzodiazepine use	72 (33.2%)	12 (38.7%)	60 (32.3%)	0.48

^a^Fisher test

*AKI* acute kidney injury, *CKD* chronic kidney disease by CKD-EPI classification, *COPD* chronic obstructive pulmonary disease, *ER* emergency room

### Neurological diagnostic tests

Sixty CT scans (IQR 0–2 days), 20 MRI scans (IQR 2–7 days), 15 EEGs (IQR 1–2.5 days), and two LPs (at admission) were performed during participants’ hospital stays (Table 2, eTable 3).

**Table 2:** Neurological diagnostic tests (NDTs), clinical management and outcomes of hospitalized patients with and without documented delirium. Values are numbers (percentages) unless otherwise stated

	Delirium ^a^ (*N* = 32)	No delirium (*N* = 185)	OR	Propensity score stratified OR	Propensity score stratified OR in sensitivity analysis ^b^
**NDT**					
Any NDT	19 (61%)	48 (26%)	4.6 (2.1–10.1)*	2.7 (1.1–6.7)^*^	3.4 (1.4–8.6)*
EEG	5 (16%)	10 (5%)	3.4 (1.1–10.7)*	5.3 (1.5–18.7)^﻿*^	6.0 (1.6–22.1)*
Lumbar puncture	0	2 (1%)	-	NA	NA
Brain MRI	7 (23%)	13 (7%)	3.9 (1.4–10.7)*	4.8 (1.6–14.8)*	4.7 (1.5–14.8)*
Brain CT scan	15 (48%)	45 (24%)	3.0 (1.4–6.4)*	1.6 (0.6–4.0)	2.0 (0.8–5.1)
**Clinical management—drugs discontinuation**					
Discontinuation of any drug of interest	4 (13%)	5 (3%)	5.4 (1.3–21.2)*	4.5 (0.9–21.6)	4.1 (0.9–20.1)
Anticholinergic SE drug discontinuation	2 (6%)	2 (1%)	6.3 (0.8–46.8)	6.4 (0.8–52.9)	6.0 (0.7–49.3)
Opioid drug discontinuation	3 (10%)	6 (3%)	3.2 (0.8–13.5)	3.9 (0.8–19.2)	4.2 (0.8–21.8)
Benzodiazepine drug discontinuation	3 (10%)	4 (2%)	4.8 (1.1–22.8)*	4.6 (0.7–32.2)	6.0 (0.7–49.3)
**Clinical management—chemical restrain**					
Neuroleptic drug initiation	16 (52%)	22 (12%)	7.9 (3.4–18.3)*	4.9 (1.9–12.6)*	7.7 (2.9–20.8)*
Benzodiazepine drug initiation	6 (19%)^c^	27 (15%)	1.4 (0.5–3.8)	1.7 (0.5–6.0)	2.4 (0.7–8.6)
**Clinical management—physical restrain**					
Any physical restraint	7 (23%)	5 (3%)	10.6 (3.1–35.9)*	6.5 (1.6–25.8)*	11.3 (2.3–54.6)*
Abdominal restraint	7 (23%)	4 (2%)	13.3 (3.6–48.7)*	7.7 (1.8–32.9)*	16.0 (2.7–94.3)*
Hand restraints	4 (13%)	1 (1%)	27.4 (2.9–254)*	21.5 (2.0–233)*	19.3 (1.7–213)*
**90-day mortality and hospital readmission**					
Hospital readmission	15 (48%)	59 (32%)	2.0 (0.9–4.3)	1.9 (0.8–4.6)	2.0 (0.8–4.9)
Death	11 (35%)	33 (18%)	2.5 (1.1–5.8)*	2.1 (0.8–5.6)	1.9 (0.7–5.1)

*NDT* neurological diagnostic test, *EEG* electro-encephalography, *MRI* magnetic resonance imaging, *CT* computed tomography, *SE* side effects, *NA* not assessed

*Delirium diagnosed within 48 h of admission

*Sensitivity analysis excluding patients in the control group who had a delirium diagnosed later in their hospital stay

*4/6 patients had alcohol withdrawal

*p-value <0.05

The proportion of patient who underwent at least one NDT was higher among patients with delirium than among patients without (Table 2). This difference persisted in logistic regressions stratified by the propensity score and in the sensitivity analysis that excluded 13 patients who had delirium diagnosed later in their hospital stay (Table 2). The characteristics of these 13 patients were similar to those of patients with delirium diagnosed at admission (eTable 4).

Higher proportions of patients with delirium also underwent each individual NDT (Table 2), and 14 (45.2%), two (6.5%), and three (9.7%) of them underwent one, two, and three NDTs, respectively. Among patients without delirium, 31 (16.7%) had one, 12 (6.5%) had two, and 5 patients (2.7%, *p* <0.001) underwent three NDTs.

Of the 67 patients who underwent at least one NDT, 37 (55.2%) showed at least one abnormal result (acute and non-acute changes) (Table 3). The result was classified as acute for 14/67 (20.9%) and helped in the diagnosis and changed the patient’s treatment management: admitted to an ICU/ stroke unite (nine patients), new drugs initiated specifically for the neurological condition (three), transferred to a tertiary hospital center (one), and/or surgery (two). None of the diagnostic yields of any of the NDTs for patients with and without delirium was statistically different (Table 3, eTable 5). Among patients who underwent NDTs, abnormal clinical neurological examination (31.6% versus 36.2%, *p* = 0.78), pre-admission falls (11.1% versus 32.6%, *p* = 0.12), and in hospital neurological consultation (34.0% versus 33.3%, *p* = 0.99) were not different between patients with and without delirium (eTable 3).

**Table 3:**
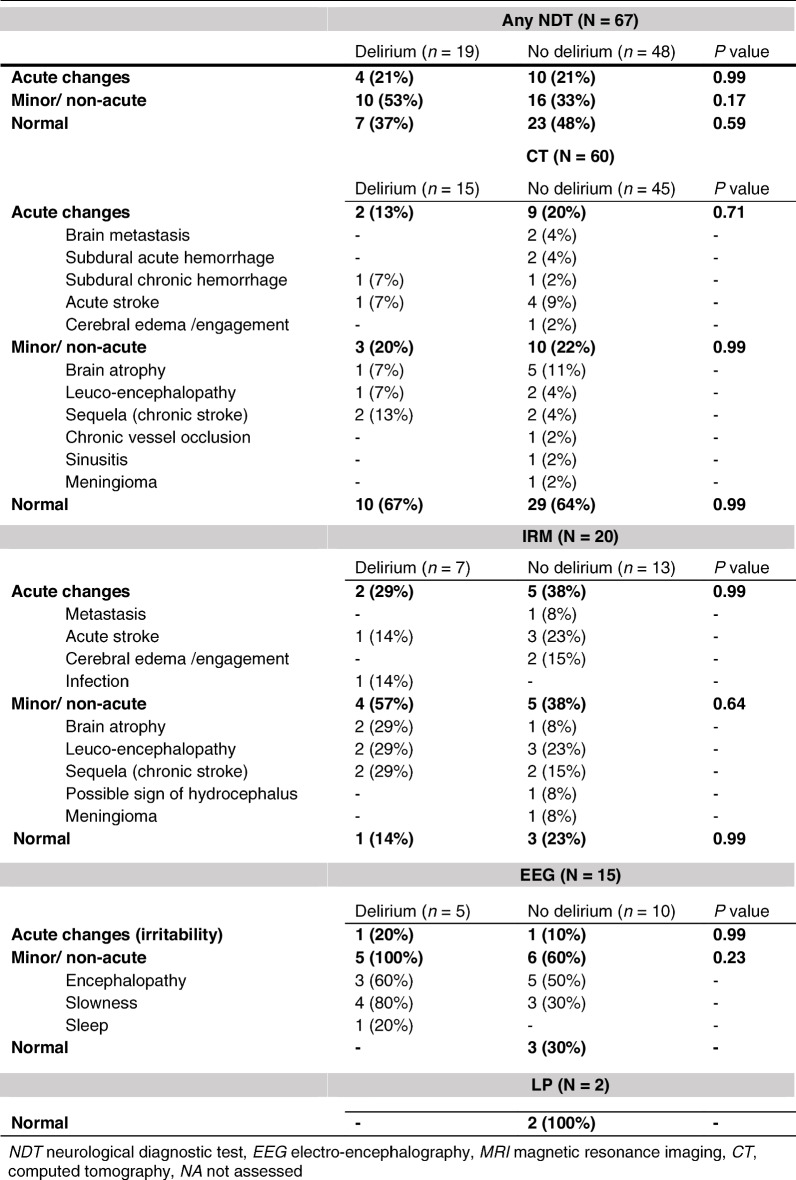
Diagnostic yield of the neurological diagnostic tests. The NDT results were classified as normal, minimal/non-acute (not affecting the diagnosis), and acute changes that impacted the patient’s management. Values are numbers (percentages) unless otherwise stated

### Blood samples, drug discontinuation, and medical/physical restrain

Patients with delirium gave a median of 4 (IQR 3–7) blood samples during their first week of hospitalization, which was not statistically different from the 5 (IQR 3–7, *p* = 0.799) given by patients without delirium.

The higher proportion of drug discontinuation (opioids, benzodiazepines, or medication with anticholinergic side effects) among patients with delirium than among patients without delirium was not statistically significant in the propensity score stratified models (Table 2).

Associations between delirium and new prescriptions of neuroleptics and between delirium and physical restraint, persisted in adjusted models (Table 2).

### Hospital length of stay and 90-day hospital readmission and mortality

Mean hospital LOS for patients with delirium (11 days, IQR 8–18) was greater than patients without delirium (8 days, IQR 5–13, *p* = 0.03). The association was not statistically significant in a log-transformed linear regression stratified by the propensity score.

Mortality was higher among patients with delirium than among patients without delirium, but the association was not statistically significant in stratified models (Table 2).

## Discussion

Nearly two-thirds of patients hospitalized with delirium undergo neurological diagnostic tests. This is a higher proportion of examination than is done for patients without delirium, but it delivers a (similar) low diagnostic yield. Overall, only 21% of all the NDTs performed among patients with and without delirium provided results that affected their treatment management. This observation highlights the need for more restrictive, evidence-based delirium workup guidelines that include multimorbid patients.

A CT scan was the most frequent test in case of delirium, but it had the lowest diagnostic yield (13% positive results versus 20% with an EEG and 21% with MRI). However, MRI and an EEGs are often performed as second-line NDTs, after an (abnormal) CT scan. Of note, the diagnostic yield of CT scans was higher among patients without delirium (20% versus 13%). This observation agrees with previously reported low proportions of positive CT scans among patients with delirium, ranging from 2.7 to 14.5% [14, 27–30]. Guidelines recommend that brain imaging should be considered for patients with new focal neurological signs, a reduced level of consciousness (not adequately explained by another cause), a history of recent falls, head injury, or anticoagulation [16]. Besides, patients who have a fever or are dehydrated, but have no focal neurological abnormalities have a 96% probability of having a normal radiological examination [29]. Nevertheless, Huschmidt et al. found that only half of patients with delirium and abnormal brain imaging had clinical signs that predicted a focal pathology [29]. Thus, patients on an anticoagulant treatment and those with persistent delirium after the resolution of precipitating factors may require additional neuroimaging [30].

Although all the EEGs of patients with delirium had minimal or non-acute changes, only one helped in patient diagnosis and management. Normal routine EEGs and continuous EEGs make delirium very unlikely [31]. Besides, EEGs show qualitative or quantitative alterations in delirium, such as marked diffuse slowing, triphasic waves, increased beta activity, occipital slowing, excess delta or theta, anteriorization, and loss of reactivity [6, 31]. However, an EEG cannot help to differentiate between underlying etiologies [31]. An EEG is important to exclude non-convulsive status epilepticus [2, 32, 33], which can be found among 7–28% of patients with confusion of unknown origin [32–35]. According to the Scottish Intercollegiate Guidelines Network, an EEG should be considered when there is a suspicion of epileptic activity or non-convulsive status epilepticus as a cause of a patient’s delirium [16]. Oh et al. suggested that an EEG may be useful for patients with a known history of seizures, findings suggestive of seizures, a history of brain trauma or stroke, or undergoing treatment with medications that lower seizure thresholds (e.g., fluoroquinolones, bupropion) [2].

No LPs were performed on patients with delirium. An LP is a minimally invasive procedure, but it is not easy to perform and may contribute to worsening the patient’s confusion [16]. There is also a risk of adverse events such as puncture site infection, cerebrospinal fluid leakage, epidural hematoma, or post-procedural headaches [36]. In one retrospective study, none of the LPs performed to rule out nosocomial meningitis in medical inpatients who had developed delirium and/or a fever was positive [37]. In a 1980s study of patients with delirium and fever, the etiology of 80 of 81 patients was not a central nervous system infection (LP were culture negative) [15].

Delirium represents a significant burden on health-care systems, with estimated cost ranging from USD 38–152 billion annually in the USA [38]. Although most of the excess cost is due to increased hospital LOS from 2.5 to 10.4 days [39], NDTs also contribute to the bill. One large Japanese study found overall diagnostic and imaging costs to be 23 and 19% higher, respectively, among patients with delirium than among patients without [40]. Many good reviews have been published [2, 11, 41], and more than 20 clinical guidelines exist on delirium prevention and management [42]. However, systematic review concluded that guidelines (many retrieved from the grey literature) were of varying quality and implementation studies remain scarce [42]. To the best of our knowledge, there have been no randomized studies testing a restrictive, criteria-based NDT approach versus usual care in work-ups for delirium using diagnostic and/or diagnostic failure as an outcome. The lack of uniform, evidence-based, good practice guidelines is a missed opportunity to avoid costly, risky, and often unnecessary tests.

The overall delirium management observed in our study was in line with recommendations [16–20]. Patients with delirium had more often medication at risk discontinuation (13% vs. 3%) compared to patients without delirium, and most had new psychotropic medication initiated (mainly neuroleptics). Benzodiazepines were given for alcohol withdrawal, and to a few patients with psychiatric comorbidities. Although we found no significant LOS, mortality or readmission differences after adjustment, larger studies have demonstrated clear adjusted associations between delirium and mortality, LOS, cognitive decline, and other unfavorable outcomes [8–10]. More than 20% of patients with delirium were managed using physical restraint and half received a new neuroleptic medication, in order to protect others (agitation) and themselves (falls, self-harm). There is scarce literature addressing the use of physical restraint in acute inpatient delirium. In a recent multicenter cross-sectional study [43], the rate of use of at least one restraint over a one-month period was 8.7% in an acute care setting. The main reason for restraint was to prevent falls (43.8%), followed by confusion or delusional behavior (20.4%) [43]. A 2003 systematic literature review highlighted the potential danger of physical restraints, with the increased risk of death or prolonged hospitalization [44].

Our study had a few limitations. First, it was a single-center study in a single department; therefore, the outcomes could be different in another setting. Patients presenting with delirium had more comorbid conditions, probably contributing to the higher number of examinations performed. Although we accounted for this in the propensity score analysis, confusion effects might come from unmeasured factors. The choice of the type and number of examinations to be performed on patients was left to the discretion of ward physicians. Although this gave us a better description of the actual practices involved in a delirium workup in a hospital setting, we cannot explicitly know which criteria informed decision to perform or not to perform each examination.

The higher proportion of neurological diagnostic tests performed on patients with delirium was associated with a low diagnostic yield, not dissimilar from the diagnostic yield of NDTs performed on inpatients without delirium. Patients and clinicians need restrictive, evidence-based guidelines to help decide when to perform an NDT as part of a delirium work-up.

## Supplementary information

ESM 1 (DOCX 87 kb)
