# Supplementary material for: Neurological diagnostic tests for patients with and without delirium: a prospective observational study
Source: GeroScience. 2024 Jun 25;46(6):6383–93. doi: 10.1007/s11357-024-01246-5 (PMC11494000; doi:10.1007/s11357-024-01246-5)
Supplement: Supplementary file 1 — (DOCX 87 kb) [file 11357_2024_1246_MOESM1_ESM.docx]

Neurological Diagnostic tests in patients with and without Delirium: a prospective observational study.

Waefker N, Abid I, Montaut V, Donze J, Zender H, John G

**Appendix**

| **eTable 1:** Association between factors and delirium and/or neurological diagnostic tests (NDTs) | P 2 |
| --- | --- |
| **eTable 2**: Precipitating factors in patients with delirium. | P 3 |
| **eTable 3:** Delay of neurological diagnostic tests (NDTs), suspected neurological deficit, pre-admission falls, and neurological consultation for patients who underwent NDTs, by presence/absence of delirium. | P 4 |
| **eTable 4:** Characteristics of patients with delirium assessed at admission and delirium diagnosed later in their hospital stay. | P5-6 |
| **eTable 5:** Diagnostic yield of the neurological diagnostic tests (NDTs). | P 7 |
| **eFigure 1:** Standardised difference between patients with and without delirium for confounding factors before and after stratification by the propensity score. | P 8 |

| **eTable 1:** Association between factors and delirium and/or neurological diagnostic tests (NDTs) | | | | |
| --- | --- | --- | --- | --- |
|  | **Delirium** | | **NDT** | |
| **Characteristic** | **OR** | ***P* value** | **OR** | ***P* value** |
| Myocardial infarct | 2.2 | 0.04 | 0.6 | 0.2 |
| Atrial fibrillation | 1.4 | 0.4 | 2.0 | 0.03* |
| Stroke/TIA | 2.6 | 0.02 | 3.1 | 0.001* |
| Cognitive impairment | 4.6 | 0.002 | 3.6 | <0.001* |
| Oncological disease | 0.6 | 0.4 | 0.5 | 0.04* |
| AKI | 2.3 | 0.03 | 0.7 | 0.4 |
| CKD | 0.6 | 0.02 | 0.8 | 0.5 |
| Psychiatric illness | 2.2 | 0.04 | 1.5 | 0.2 |
| Rhumatological disease | 2.5 | 0.02 | 1.6 | 0.1 |
| Malnutrition | 3.2 | 0.003 | 1.4 | 0.3 |
| Urinary incontinence | 1.8 | 0.1 | 2.1 | 0.02* |
| Nocturia | 0.3 | 0.004 | 0.8 | 0.4 |
| Urinary catheter | 4.3 | 0.002 | 2.3 | 0.04* |
| Surgery within the month | 4.9 | 0.01 | 1.6 | 0.4 |
| Pre-admission neuroleptic | 2.9 | 0.01 | 2.1 | 0.04* |
| Pre-admission antidepressant | 3.5 | 0.002 | 1.3 | 0.5 |
| * Included in the propensity score  NDT: neurological diagnostic test; AKI, acute kidney injury; CKD, chronic kidney disease by CKD-EPI classification; TIA, transient ischemic attack. | | | | |

| **eTable 2**: Precipitating factors in patients with delirium (N=32). | |
| --- | --- |
| **Factor** | **n (%)*** |
| Infection | 23 (72%) |
| Trauma-Pain | 3 (9%) |
| Drugs – (opioids, benzodiazepines, other) | 11 (35%) |
| Urinary retention | 0 |
| Faecal impaction | 1 (3%) |
| ICU | 7 (22%) |
| Primary neurological disease | 2 (6%) |
| Stroke | 3 (9%) |
| Head trauma - neurosurgery | 2 (6%) |
| Toxic, metabolic | 5 (16%) |
| Alcohol, drugs withdrawal | 4 (13%) |
| Epilepsy | 3 (9%) |
| Surgery | 5 (16%) |
| * Multiple precipitating factors are often found in patients with delirium. | |

| **eTable 3:** Delay of neurological diagnostic tests (NDTs), suspected neurological deficit, pre-admission falls, and neurological consultation for patients who underwent NDTs, by presence/absence of delirium. | | | |
| --- | --- | --- | --- |
|  | **Patients with delirium**  **Median in days (IQR)*** | **Patients without**  **Median in days (IQR)*** | ***P* value** |
| **Brain CT scan** | 0 (0-2.5) | 0 (0-2) | 0.54 |
| **EEG** | 2 (1-3) | 1.5 (0-2) | 0.45 |
| **LP** | - | 0 (NA) | NA |
| **MRI** | 4 (2-10) | 4 (1-6) | 0.57 |
|  |  |  |  |
|  | **With delirium (N=19)**  **n (%)** | **Without delirium (N=48)**  **n (%)** |  |
| **Abnormal neurological exam**** | 6 (32%) | 17 (36%) | 0.72 |
| Palsy | 2 (11%) | 8 (17%) | 0.71 |
| Gait/balance problem | 1 (5%) | 4 (8%) | 0.99 |
| Speech problem / aphasia | 2 (11%) | 2 (4%) | 0.57 |
| Epilepsy | 1 (5%) | 5 (11%) | 0.66 |
| **Pre-admission falls** | 2 (11%) | 15 (33%) | 0.12 |
| **Any above** | 8 (44%) | 30 (65%) | 0.16 |
| **Non above** | 10 (56%) | 16 (35%) | 0.16 |
|  |  |  |  |
| **Any Neurological consultation** | 6 (33%) | 16 (34%) | 0.99 |
| IQR: interquartile range; NA: not assessed  *Days elapsed between hospital admission and neurological diagnostic tests performed; ** abnormal neurological exam do not include presence of delirium | | | |

| **eTable 4:** Characteristics of patients with delirium assessed at admission and delirium diagnosed later in their hospital stay. | | | | |
| --- | --- | --- | --- | --- |
| **Characteristic** | **Cohort**  **(N = 217)** | **Delirium at admission (N = 32)** | **Delirium during hospitalization**  **(N = 13)*** | ***P* value** |
| Age (y), median (IQR) | 75.9 (66.5-85.8) | 84.0 (73.2-90.7) | 77.4 (71.7-81.5) | 0.18 |
| Male | 109 (50.2%) | 11 (35.5%) | 7 (53.8%) | 0.32 |
| Admitted through ER | 205 (94.5%) | 31 (100%) | 12 (92.3%) | 0.29 |
| None smoker  Current somker  Former smoker | 152 (70.0%)  43 (19.8%)  22 (10.1%) | 24 (77.4%)  5 (16.1%)  2 (6.4%) | 11 (84.6%)  2 (15.4%)  0 (0%) | 0.99 |
| Charlson comorbidity index, median (IQR) | 3 (2-5) | 4 (3-6) | 2 (1-4) | 0.01 |
| High blood pressure | 140 (64.5%) | 23 (74.2%) | 11 (84.6%) | 0.70 |
| Myocardial infarct | 53 (24.4%) | 12 (38.7%) | 1 (7.7%) | 0.07 |
| Atrial fibrillation | 63 (29.0%) | 11 (35.5%) | 4 (30.8%) | 0.99 |
| Peripheral vascular disease | 19 (8.8%) | 2 (6.5%) | 1 (7.7%) | 0.99* |
| Heart failure | 90 (41.5%) | 16 (51.6%) | 5 (38.5) | 0.52 |
| Stroke | 43 (19.8%) | 11 (35.5%) | 3 (23.1%) | 0.50 |
| Cognitive impairment  Mild  severe | 44 (20.3%)  39 (18.0%) | 11 (35.5%)  11 (35.5%) | 1 (7.7%)  4 (30.8%) | 0.08 |
| Diabetes  Insulin  No insulin | 49 (22.6%)  20 (9.2%)  29 (13.4%) | 7 (22.6%)  3 (9.7%)  4 (12.9%) | 1 (7.7%)  1 (7.7%)  0 (0%) | 0.58 |
| COPD | 40 (18.4%) | 4 (12.9%) | 1 (7.7%) | 0.99 |
| Oncological disease  Without metastasis  With distant metastasis | 40 (18.4%)  26 (12.0%) | 6 (19.3%)  1 (3.2%) | 2 (15.4%)  2 (15.4%) | 0.43 |
| Mild liver disease  Severe liver disease | 17 (7.8%)  13 (6.0%) | 2 (6.5%)  1 (3.2%) | 0 (0%)  1 (7.7%) | 0.77 |
| AKI  1  2  3 | 53 (24.4%)  10 (4.6%)  5 (2.3%) | 10 (32.3%)  2 (6.4%)  3 (9.7%) | 4 (30.8%)  1 (7.7%)  0 (0%) | 0.85 |
| CKD 0  I  II  III  IV  V | 162 (74.7%)  1 (0.5%)  5 (2.3%)  34 (15.7%)  15 (6.9%)  - | 18 (58.1%)  0 (0%)  1 (3.2%)  9 (29.0%)  3 (9.7%)  - | 12 (92.3%)  0 (0%)  1 (7.7%)  0 (0%)  0 (0%)  - | 0.05 |
| Rheumatological disease | 78 (35.9%) | 17 (54.8%) | 4 (30.8%) | 0.52 |
| Anemia | 80 (36.9%) | 11 (35.5%) | 6 (46.1%) | 0.86 |
| Psychiatric illness | 71 (32.7%) | 15 (48.4%) | 3 (23.1%) | 0.18 |
| Alcohol Consumption  Active  Former | 38 (17.5%)  17 (7.8%) | 4 (12.9%)  1 (3.2%) | 3 (23.1%)  3 (23.1%) | 0.06 |
| Drug abuse  Active  Former | 9 (4.1%)  4 (1.8%) | 1 (3.2%)  0 (0%) | 8 (4.3%)  4 (2.1%) | 0.51 |
| Malnutrition  Mild  Moderate  Severe | 24 (11.1%)  31 (14.3%)  25 (11.5%) | 5 (16.1%)  7 (22.6%)  7 (22.6%) | 0 (0.0%)  2 (15.4%)  4 (30.7%) | 0.46 |
| Nasal tube feeding | 8 (3.8%) | 3 (10.3%) | 1 (8.3%) | 0.99 |
| Urinary incontinence | 48 (22.1%) | 10 (32.3%) | 4 (30.8%) | 0.99 |
| Nocturia | 104 (47.9%) | 7 (22.6%) | 2 (15.4%) | 0.70 |
| Urinary catheter | 25 (11.5%) | 9 (29.0%) | 2 (15.4%) | 0.46 |
| Surgery within the month | 12 (5.5%) | 5 (16.1%) | 2 (15.4%) | 0.99 |
| Pre-admission diuretics use | 88 (40.5%) | 13 (41.9%) | 3 (23.1%) | 0.31 |
| Pre-admission opioids | 59 (27.2%) | 9 (29.0%) | 4 (30.8%) | 0.99 |
| Pre-admission neuroleptic use | 36 (16.6%) | 10 (32.3%) | 4 (30.8%) | 0.99 |
| Pre-admission antidepressant use | 49 (22.6%) | 14 (45.2%) | 2 (15.4%) | 0.09 |
| Pre-admission benzodiazepine use | 72 (33.2%) | 12 (38.7%) | 4 (30.7%) | 0.74 |
| * Delirium at admission was diagnosed through a systematic exam by a neuropsychologist, whether delirium diagnosed later during hospital stay was based on clinical evaluation of the ward physician.  AKI, acute kidney injury; CKD, chronic kidney disease by CKD-EPI classification; COPD, chronic obstructive pulmonary disease; ER, emergency room. | | | | |

| **eTable 5:** Diagnostic yield of the neurological diagnostic tests (NDTs). Values are numbers (percentages) unless otherwise stated. | | | | | |
| --- | --- | --- | --- | --- | --- |
|  | **Number of exams with minimal / non acute changes*** | **Number of exams with acute changes** ^†^ | | | |
|  |  | **Cohort** | **With delirium** | **Without delirium** | ***P* value** |
| **Any NDT** | 28/67 (41.8%) | 14/67 (20.9%) | 4/19 (21.0%) | 10/48 (20.8%) | 0.98 |
| **Brain CT-scan** | 10/59 (17.0%) | 11/59 (18.6%) | 2/15 (13.3%) | 9/44 (20.5%) | 0.54 |
| **Brain MRI** | 11/20 (55.0%) | 7/20 (35%) | 2/7 (28.6%) | 5/13 (38.5%) | 0.66 |
| **EEG** | 13/15 (86.7%) | 3/15 (20.0%) | 1/5 (20.0%) | 2/10 (20.0%) | 0.99 |
| **Lumbar puncture** | 0 | 0 | - | 0 | NA |
| NDT: neurological diagnostic test; EEG: electro-encephalography; MRI: magnetic resonance imaging; CT: computed tomography; NA: not assessed.  The abnormal results of NDTs were classified as:  * minimal/not acute changes (e.g. brain atrophy in MRI or CT)  † acute changes that helped in the diagnosis or impacted the patient’s treatment management (e.g. acute stroke, subdural hematoma on images, focal electrical activity on EEG). | | | | | |


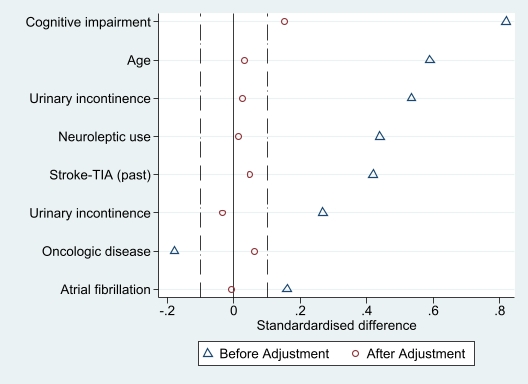


**eFigure1:** Standardised difference between patients with and without delirium for confounding factors before and after stratification by the propensity score. The highly unequal repartition of factors before stratification are adequately balanced after stratification.
